# Differences in and Prognostic Value of Quality of Life Data in Rectal Cancer Patients with and without Distant Metastases

**DOI:** 10.3390/healthcare9010001

**Published:** 2020-12-22

**Authors:** Fabian Frank, Markus Hecht, Florian Loy, Sandra Rutzner, Rainer Fietkau, Luitpold Distel

**Affiliations:** Department of Radiation Oncology, Universitätsklinikum Erlangen, Friedrich-Alexander-Universität of Erlangen-Nürnberg (FAU), D-91054 Erlangen, Germany; fabian.frank@fau.de (F.F.); markus.hecht@uk-erlangen.de (M.H.); Flo-loy@web.de (F.L.); Sandra.rutzner@uk-erlangen.de (S.R.); rainer.fietkau@uk-erlangen.de (R.F.)

**Keywords:** colorectal cancer, metastatic disease, patient reported outcomes, health related quality of life, prognostication

## Abstract

(1) Background: Individualization of treatment is a major challenge in oncology and requires a variety of predictive and prognostic parameters. In addition to tumor biology analyses, baseline health-related quality of life might be a valid tool to predict overall survival. This study was conducted to evaluate the prognostic relevance of baseline quality of life data in patients with rectal cancer. In this context, differences between patients with and without distant metastases were of particular interest. (2) Methods: Our cohort included 258 patients with rectal cancer treated in the radiotherapy department of the University Hospital Erlangen. Patients completed the European Organisation for Research and Treatment of Cancer (EORTC) core quality of life questionnaire (QLQ C30) and colorectal cancer questionnaire (CR38). Clinical and survival data were provided by the Gießener Tumor Documentation System (GTDS) of the Comprehensive Cancer Center Erlangen-EMN (CCC, Friedrich-Alexander University Erlangen-Nuremberg, Erlangen, Germany). Statistical analyses were performed using Kaplan–Meier analyses and univariate and multivariate Cox regression. (3) Results: A cohort of 258 patients with rectal adenocarcinoma was analyzed including 50 patients (19.4%) with metastatic disease. No differences were observed between patients with and without distant metastases in most areas of quality of life studied, with the exception of physical function, loss of appetite, chemotherapy side effects and weight loss. Gender, baseline physical function, sexual function, diarrhea, and weight loss over time had a prognostic value in the entire cohort. Appetite loss was an additional prognostic parameter in patients with distant metastases. (4) Conclusions: The quality of life of patients with metastatic disease differed only slightly from non-metastatic patients. Health-related quality of life data provide prognostic information for patients with rectal cancer.

## 1. Introduction

Health-related quality of life (HRQoL) is a concept that has gained increasing importance in cancer research in recent years. Both as a valid endpoint in studies [1,2,3] and as a prognostic tool itself [4,5,6]. Overall survival (OS) has long been considered as the most relevant endpoint in cancer studies. Over time, other tumor-related outcomes such as progression-free survival or disease-free survival have been introduced, which are not necessarily of critical importance to the patients themselves [7]. Patients provide a relevant perspective on their own disease, quality of life, and symptom burden. Studies have concluded that assessment of these patient-reported outcomes (PROs) is more accurate than external measurement by physicians or other health professionals [8]. The prognostic implications of HRQoL data have been widely reported for colorectal cancer [4,5,9,10,11] and other cancer entities, including head and neck [12], breast [13,14,15], and brain [16]. Standard therapy for rectal carcinoma includes neoadjuvant radiochemotherapy [17,18]. This therapy is locally very efficient and thus leads to a local cure. Unfortunately, 20% to 35% of patients have metastatic disease at the time of diagnosis [19,20] and 20% up to 50% of patients develop metastases during the course of the disease [21]. These patients have a very poor prognosis. Patients with metastatic disease have a five-year survival of 13.1% compared to 90.1% for non-metastatic patients [22]. The aim of this study was to investigate HRQoL as a potential predictor of OS in our patient cohort. It was of interest to investigate whether patients with metastatic disease suffered more than patients who were cured by a treatment regimen including neoadjuvant radiochemotherapy.

## 2. Materials and Methods

### 2.1. Patients

This open cohort study combines quality of life data collected consecutively between 2005 and 2017 at the radiotherapy department of the University Hospital Erlangen. Data collection was prospective and a total of 258 patients with rectal cancer were included. Inclusion criteria were confirmed rectal cancer diagnosis, treatment with combined radio-chemotherapy and written informed consent to participate. All patients received systemic chemotherapy and were treated with 50.4 Gy of ionizing radiation. Data on clinicopathologic factors, including TNM Classification of Malignant Tumors (TNM), Union internationale contre le cancer (UICC) classification, chemotherapy, surgery, radiation, and vital status were obtained by the Comprehensive Cancer Center Erlangen-EMN. Missing clinical data were collected from electronic medical records. Demographic and basic disease characteristics are listed in Table 1.

### 2.2. Treatment

The radiation treatment regimen was a four-field box technique with three-dimensional conformal radiotherapy. Patients were treated with daily doses of 1.8 Gy up to a total dose of 50.4 Gy, 30 patients also received hyperthermia. One hundred and forty-nine nonmetastatic patients received neoadjuvant treatment, 12 received adjuvant treatment, and 22 were not surgically treated. After completion of radiochemotherapy, patients were treated with a total mesorectal resection of the cancer. The most commonly used concurrent chemotherapy combination was 5-FU and oxaliplatin. The remaining patients received similar treatment regimens including 5-FU solo, 5-FU + capecitabine, 5-FU + antibody, 5-FU + cisplatin or 5-FU + irinotecan. Metastatic patients typically received FOLFOX, FOLFIRI or FOLFOX-IRI, in some cases in combination with antibodies.

### 2.3. Quality of Life

Quality of life data were prospectively collected using the EORTC QLQ C30 [23] and EORTC QLQ CR38 [24] questionnaires at various time points throughout the therapy. For this study, the baseline score obtained immediately before therapy and the post-treatment score obtained in the first week after the end of therapy were considered. The EORTC QLQ C30 consists of 30 items and assesses oncological patients multidimensionally over 10 scores. Functional scores are physical, role, cognitive, and emotional function. Symptom scores are pain, fatigue, nausea, and vomiting. Other scores are global quality of life and various derived from single items: dyspnea, insomnia, appetite loss, constipation, diarrhea, and financial difficulties. Higher values in the functional scores indicate better quality of life, while higher values in the symptom scores indicate more symptoms and thus a lower quality of life. The EORTC QLQ CR38 consists of 38 items covering symptoms and side-effects related to various treatment modalities, body image, sexuality, and future perspective. All QoL scores were calculated according to the official EORTC manuals.

### 2.4. Statistics

Unpaired *t*-tests were performed for all functional, symptom, and rectal specific scores to detect differences between metastatic and non-metastatic patients. Levene’s test for equal variance and Cohen’s D were calculated for each variable. Significant findings were analyzed as described below. To check other staging variables T and N status were examined in the same method.

### 2.5. Survival Analysis

The clinical outcome considered in this trial was overall survival, defined as time until tumor related death. In the absence of death confirmation OS was censored at the date of last contact. Univariate and multivariate Cox regression models were used for the analysis. The variables used were QoL functional scores, QoL symptom scores, QoL rectum specific scores, and gender. First, the risks for the quality of life indicated prior to therapy were calculated and defined as “baseline scores”. Hazard ratios for baseline QoL data were calculated for every 20% change [25]. Changes between baseline and post-treatment QoL assessments were calculated as dichotomized variable (deterioration yes or no) and defined as “change scores”. All Cox regressions were calculated age adjusted. First univariate Cox regression was performed with significance level of 0.05. All identified variables were then tested in multivariate Cox regression. In a first block, age and gender integrated into the model using the enter method. In a second block, identified variables were then tested as stepwise backwards model with entry and removal levels of 0.05 and 0.10, respectively. The proportional hazards assumption was tested by visual inspection of the log-minus-log curves and was found to be satisfactory for all multivariate covariates. Kaplan–Meier survival plots were used for survival estimation and compared using the log rank test. For balanced group creation, baseline variables were split at median [11]. All statistical analysis was performed with SPSS 26 (IBM, Armonk, NY, USA).

## 3. Results

Due to low response rate female sexual problems (missing 254 of 258), stoma problems (missing 225 of 258), and sexual satisfaction (missing 189 of 258) were not included in the analysis.

### 3.1. Baseline Characteristics

**Figure 1:**
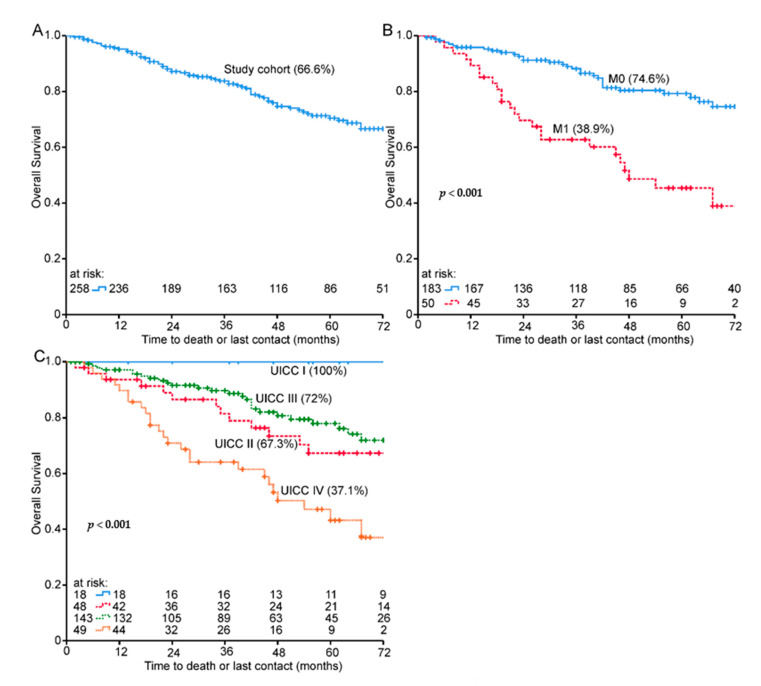
Kaplan–Meier plots for (**A**) overall survival of the entire cohort, (**B**) TNM: distant metastasis M and (**C**) stage UICC I–IV. Seventy-two-month overall survival % in brackets.

A total of 258 patients with rectal cancer were included. The median follow-up time was 64.4 months (range 12–152 months). Overall survival for all patients was 66.6% at 72 months (Figure 1A). Patients had mainly advanced cancer (T3 stage 162/62.8%) with affected lymph nodes (N1 stage 135/52.3%) and distant metastasis (M1 stage 50/19.4%) (Table 1). The median age was 67.4 years and 74% of patients were male.

### 3.2. Patients Suffering from Metastases

**Figure 2:**
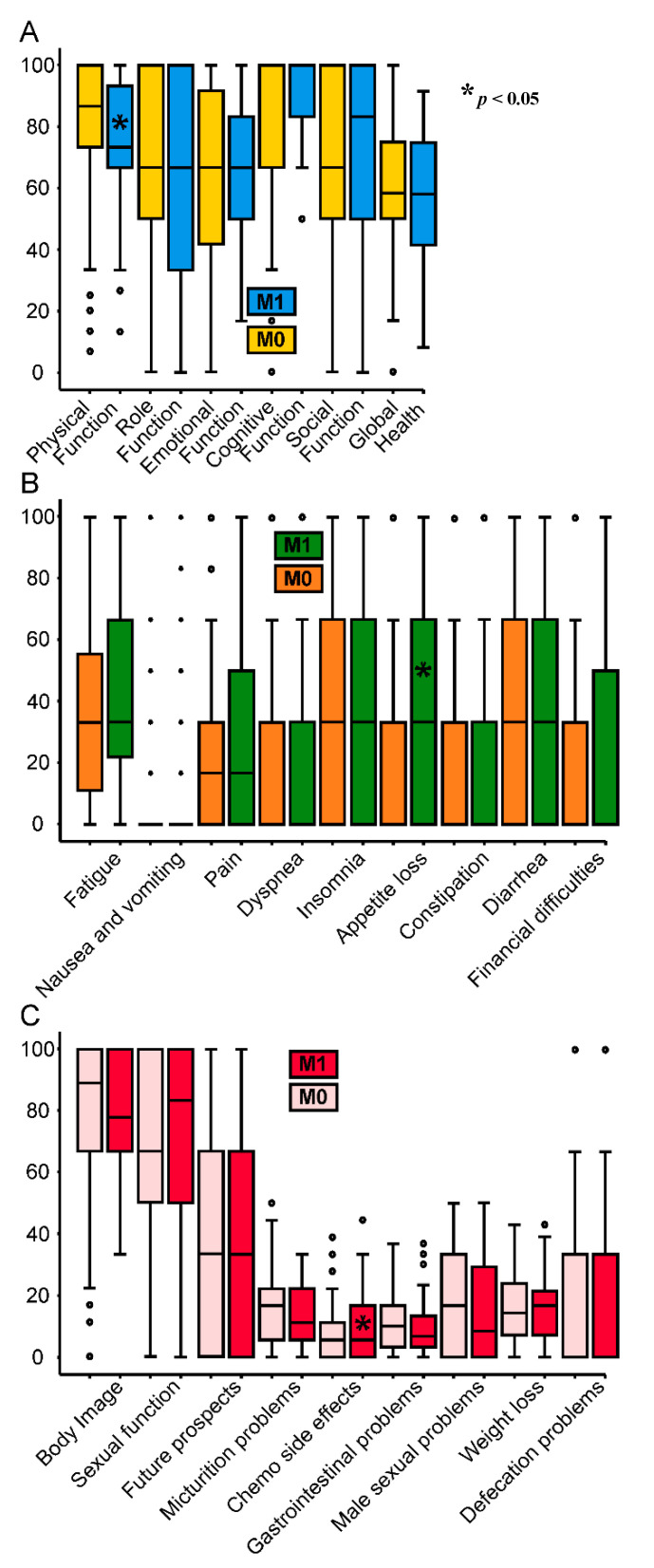
Side-by-side box plots M0 (*n* = 183) vs. M1 (*n* = 50) of baseline (**A**) QLQ C30 functional scores M0 (yellow), M1 (blue), (**B**) QLQ C30 symptom scores M0 (orange), M1 (green), (**C**) QLQ CR38 scores M0 (pale pink), M1 (red), * marks domains significant in an unpaired *t*-test.

In our cohort, 50 patients (19.4%) had a metastatic disease. Metastatic status increased the relative hazard of dying by 281% (95% CI = 2.25–6.47, *p* < *0*.001). Overall survival at 6 years was 74.6% in the M0 group compared to 38.9% in the M1 group. (Figure 1C). Box plots of the individual EORTC domains are presented in Figure 2. Most scores of patients who had a metastatic disease were not different from patients with cured rectal cancer. Physical function, appetite loss, chemo side effects for baseline scores, and weight loss for change scores differed significantly by unpaired *t*-test (Table 2 marked with * in Figure 2). In addition, patients with tumor stage greater than or equal to T3 showed significant differences in some categories, namely nausea and vomiting (mean difference + 4.58), future prospect (mean difference + 13,6), and defecation problems (mean difference + 4,4). It should be noted that 44 of 50 metastatic patients were also in this group. Positive N status did not result in a significant difference in any HRQoL variable.

**Figure 3:**
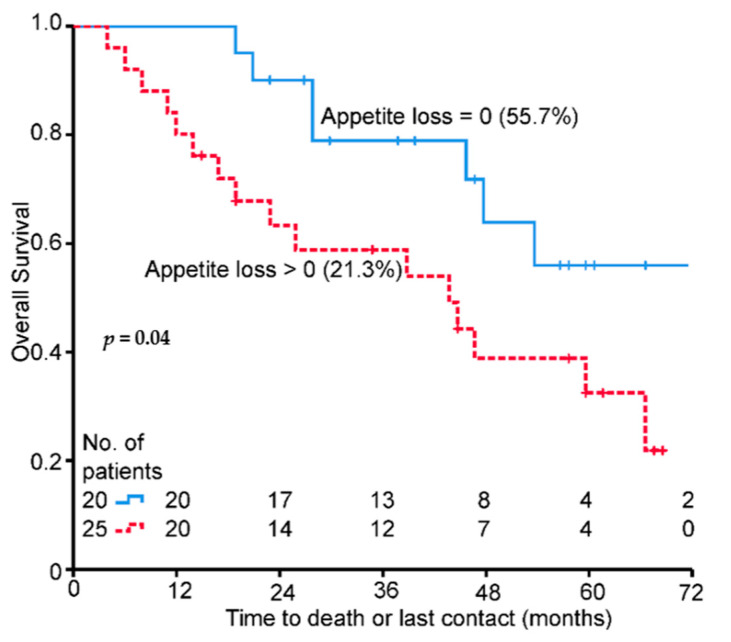
Baseline score Kaplan–Meier plots for overall survival and appetite loss, M1 patients only, 72-month OS% in brackets.

**Figure 4:**
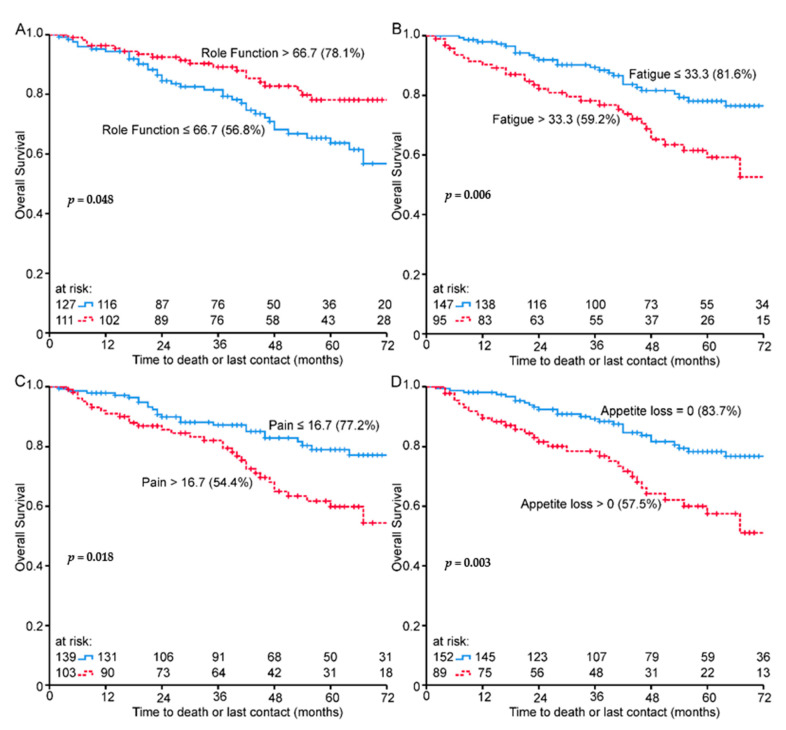
Baseline scores Kaplan–Meier plots for overall survival and (**A**) role function, (**B**) fatigue, (**C**) pain and (**D**) appetite loss, 72-month OS% in brackets.

Appetite loss is the only QoL score of patients with metastases that is clearly associated with survival in both univariate and multivariate analyses (Table 3). The Kaplan–Meier plot for appetite loss in the metastatic patients (Figure 3) shows similar results to those of the entire cohort (Figure 4D).

### 3.3. Prognostic Value of Baseline EORTC QLQ C30 and CR38 Data in the Whole Cohort

Cox regression analyses were performed for baseline quality of life (Table 4). In univariate analysis physical function, role function, fatigue, nausea and vomiting, pain, dyspnea, appetite loss, male sexual problems, and weight loss were significantly associated with survival. Global health score was not associated with survival. All of these hazard ratios indicate a higher survival for patients with higher functional scores and shorter survival for patients with higher symptom scores. In multivariate analysis, physical function clearly remained a favorable influential factor as did gender. Male patients had a 46% higher risk of death. The hazard ratio for physical function indicates that for every 20% increase, the relative hazard of dying decreased by 29% (95% CI = 0.57–0.88). The other variables remained nonsignificant in the multivariate model. Kaplan–Meier survival plots display differences in overall survival between groups for role function (*p* = 0.048) (Figure 4A), fatigue (*p* = 0.006) (Figure 4B), pain (*p* = 0.018) (Figure 4C), and appetite loss (*p* = 0.003) (Figure 4D).

### 3.4. Prognostic Value of Change EORTC QLQ C30 and CR38 Data in the Whole Cohort

**Figure 5:**
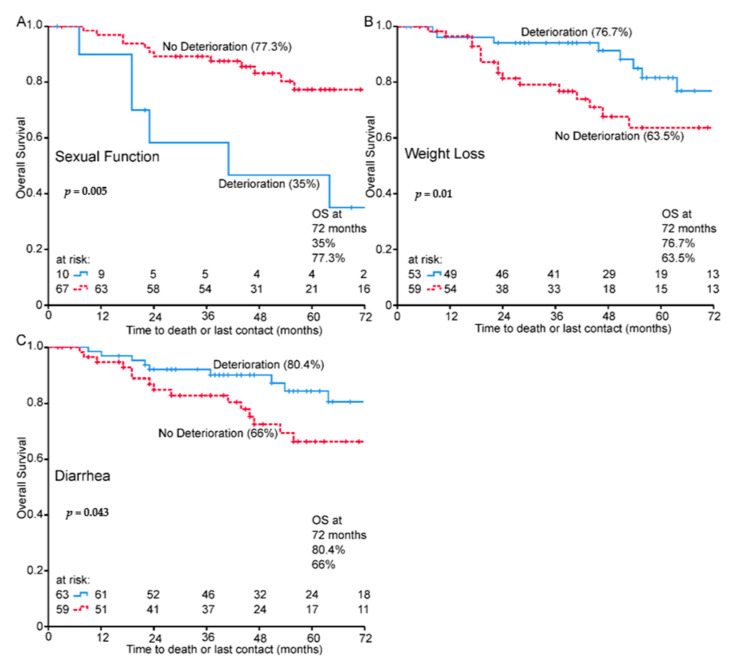
Change scores treatment Kaplan–Meier charts for overall survival and (**A**) sexual function, (**B**) weight loss and (**C**) diarrhea, 72-month OS% in brackets.

Cox regression analysis was performed for change scores (Table 5). In univariate analysis diarrhea, sexual function, and weight loss were significant for survival. Age-adjusted multivariate analysis identified diarrhea (HR = 0.34, 95%CI = 0.128–0.93, *p* = 0.035), sexual function (HR = 3.13, 95% CI = 1.16–8.46, *p* = 0.024), and weight loss (HR = 0.26, 95% CI = 0.09–0.79, *p* = 0.017) as significant predictors of survival. HRs indicates worse survival for deterioration in sexual function and longer survival for worsening in diarrhea and weight loss. Kaplan–Meier survival plots show differences between groups for sexual function (Figure 5A), weight loss (Figure 5B) and diarrhea (Figure 5C).

## 4. Discussion

This study investigated the prognostic value of EORTC QLQ C30 and CR38 data for patients with rectal cancer. A particular focus was on patients with metastases who performed slightly better in overall survival than expected. Five year overall survival is reported with 62% in Germany [26], whereas our cohort had 70.5%.

The metastatic patients had little to no significant difference in most QoL domains. To the best of our knowledge, this has not been previously reported. They differed in only four domains. Baseline scores for physical function, loss of appetite, side effects of chemotherapy, and change scores for weight loss. Regarding survival prediction, only appetite loss (HR 1.30 95% CI = 1.01–1.66, *p* = 0.043) was prognostically relevant. Metastatic status has previously been associated with worse QoL in various cancer types before [27,28]. Contrary to intuitive expectation, the bad news of metastatic disease does not seem to clearly affect QoL. Cancer diagnosis itself is associated with poorer self-reported QoL [29]. However, positive metastatic status was strongly associated with overall survival (HR 3.81, 95% CI = 2.25–6.47, *p* < *0*.001), which is consistent with previous reports [25,30,31]. Otherwise, resectable metastases are no longer associated with a significantly worse prognosis [32]. Efficace et al. [5] have shown that social function is prognostic in metastatic colorectal cancer beyond several biomedical parameters, a result we could not reproduce. The difference in HRQoL in metastatic vs. non-metastatic patients should be reproduced by other research groups. Further research is needed to confirm or reject this finding.

Regarding the predictive power of the HRQoL data for the entire cohort, we were able to support our assumptions. In univariate analysis of baseline QoL scores, physical function, role function, fatigue, nausea and vomiting, pain, dyspnea, appetite loss, male sexual problems, and weight loss were prognostic for survival. All of these factors have been described as prognostically relevant in previous studies, although sometimes in different combinations [4,5,7,11,33,34]. Physical function, role function, fatigue, pain, dyspnea, insomnia, and appetite loss have also been described as prognostic in other tumor types [15,25,30,35,36]. Multivariate analysis in our study leaves physical function and male gender as significant prognostic QoL factors. In Germany, colorectal cancer affects more men than women (10-year prevalence 196,100 and 159,500, respectively). The relative 10-year survival rate in Germany is 60% for women, but 56% for men [26]. Our results support a worse prognosis for male patients.

The change scores for sexual function, weight loss, and diarrhea were predictive for overall survival in our cohort, interestingly with a longer OS for patients with more diarrhea and more weight loss. Better sexual function has been previously described as a positive prognostic factor [37]. This study cannot provide an explanation for the unexpected association of an increase in diarrhea and weight loss with longer patient survival.

### 4.1. Strengths and Weaknesses

A homogeneous cohort with a uniform treatment regimen was studied. Valid and reliable tools were used to prospectively measure HRQoL. In addition, reliable survival data were available. The study itself relied on data that were not collected to test a specific intervention. The cohort was consecutively surveyed and mainly patients were included speaking German and therefore cannot be representative of all colorectal cancer patients. Due to nature of the HRQoL data, it is not a randomized controlled trial and therefore no causal relationships can be derived. The data were collected in a time frame around the administration of therapy, so disease- or therapy-related impairments in quality of life late in the course of the disease are not detected.

### 4.2. Comparability

A variety of correlations between HRQoL and OS have been reported over the years, but it remains difficult to identify individual predictors [9]. QoL data tend to act as surrogates for underlying prognostic factors [38]. It is undisputed that HRQoL provides valuable information. A meta-analysis of 30 randomized controlled trials from the EORTC [39] shows physical function, nausea and vomiting, pain, and appetite loss as prognostic factors in colorectal cancer that are close to our results. The persistent inconsistency of results leads to the assumption that there may not be a single predictor of OS in the HRQoL sphere. Differences in study design particularly in cohort selection, timing of QoL assessment, therapy, stage of disease, and control for other parameters make it difficult to reproduce findings [40]. However, this does not undermine the importance of QoL measurement in cancer trials.

## 5. Conclusions

Contrary to our expectations, patients with metastasis report an equally good quality of life as cured patients with rectal carcinoma. Future research is needed to confirm this finding. It should be noted that HRQoL was collected in relatively close proximity to therapy. Both baseline and change score of EORTC QLQ C30 and CRC38 provide prognostic information in patients with rectal carcinoma. Our results demonstrate the value of PROs when assessing HRQoL with EORTC questionnaires. The specific domains found to be of prognostically relevant provide emphasis areas for intervention and future trials.

## Figures and Tables

**Table 1:** Demographic and basic disease characteristics.

Risk Factor	Total No.	No. (%)
Age (years) ^1^	258	67.4 (11.6)
Gender	258	
Male		191 (74)
Female		67 (26)
Status	258	
Alive		184 (71.3)
UICC	258	
I		21 (8.1)
II		49 (19)
III		141 (54.7)
IV		47 (18.2)
TNM: cT	258	
1		6 (2.3)
2		30 (11.6)
3		162 (62.8)
4		60 (23.3)
TNM: cN	255	
X		3 (1.2)
0		70 (27.1)
1		135 (52.3)
2		50 (19.4)
TNM: cM	233	
X		25 (9.7)
0		183 (70.9)
1		50 (19.4)
TNM: pL	214	
X		44 (17.1)
0		172 (66.7)
1		42 (16.3)
TNM: pV	212	
X		46 (17.8)
0		202 (78.3)
1		10 (3.9)

^1^ For Age, No. (%) are mean (standard deviation).

**Table 2:** M0 compared to M1 for quality of life baseline and change scores using the *t*-test.

Risk Factor ^1^	*p*-Value (2-Tailed)	Mean Difference	95% CI	Cohen’s d
*Baseline*				
Physical function	0.036	7.78	0.51–15.05	0.286
Appetite loss	0.008	−15.35	−26.62–4.09	0.716
Chemotherapy Side Effects	0.015	−4.66	−8.37–0.95	0.657
*change*				
Weight loss	0.008	18.33	4.91–31.75	0.797

^1^ significant only.

**Table 3:** Overall survival of age-adjusted hazard ratios for M1 patients baseline and change scores QLQ C30 and QLQ CR38.

		Univariate		Multivariate	
Risk Factor ^1^	Deterioration	HR (95% CI)	*p*-Value	HR (95% CI)	*p*-Value
*Baseline* ^2^					
Physical function		0.70 (0.49–1.01)	0.055		
Appetite loss		1.30 (1.01–1.66)	0.043	1.30 (1.01–1.66)	0.043
Chemotherapy side effects		1.15 (0.51–2.60)	0.739		
*Change*					
Weight loss	No Yes	2.67 (0.46, 15.28)	0.272		

^1^ differing from overall collective only ^2^ HRs calculated for every 20%.

**Table 4:** Overall survival of age-adjusted hazard ratios for baseline QLQ C30 and QLQ CR38.

		Univariate		Multivariate	
Risk Factor ^1^	Mean (SD)	HR (95% CI)	*p*-Value	HR (95% CI)	*p*-Value
Gender *QLQ C30*		1.19 (0.91–1.56)	0.208	1.46 (1.07–2.01)	0.011
Physical function	79.67 (22.30)	0.70 (0.59-0.84)	<0.001	0.71 (0.57-0.88)	0.002
Role function	69.26 (30.33)	0.83 (0.71–0.97)	0.02	1.13 (0.86–1.48)	0.375
Emotional function	64.02 (25.95)	0.96 (0.79–1.16)	0.669		
Cognitive function	84.30 (21.71)	0.84 (0.67–1.06)	0.15		
Social function	66.32 (30.38)	1.02 (0.85–1.21)	0.863		
Global health	58.95 (22.30)	0.89 (0.73–1.09)	0.248		
Fatigue	36.29 (27.74)	1.32 (1.10–1.58)	0.003	0.84 (0.58–1.21)	0.351
Nausea and vomiting	6.30 (15.61)	1.36 (1.04–1.76)	0.024	1.07 (0.73–1.58)	0.732
Pain	24.79 (29.37)	1.26 (1.07–1.50)	0.004	1.11 (0.89–1.39)	0.369
Dyspnea	18.67 (27.33)	1.24 (1.03–1.50)	0.021	1.23 (0.99–1.53)	0.068
Insomnia	33.61 (33.20)	1.12 (0.95–1.32)	0.194		
Appetite loss	19.08 (28.79)	1.34 (1.14–1.58)	<0.001	1.18 (0.93–1.50)	0.171
Constipation	14.70 (28.29)	0.86 (0.68–1.10)	0.225		
Diarrhea	34.45 (36.01)	1.15 (0.97–1.34)	0.078		
Financial difficulties	21.52 (31.61)	0.99 (0.82–1.20)	0.944		
*QLQ CR38*					
Body Image	77.81 (25.78)	0.85 (0.69–1.05)	0.125		
Sexual function	71.72 (29.36)	1.11 (0.88–1.41)	0.369		
Future prospects	34.18 (33.75)	1.01 (0.85–1.18)	0.949		
Micturition problems	15.75 (10.86)	1.33 (0.83–2.13)	0.241		
Chemotherapy side effects	7.61 (9.52)	1.48 (0.79–2.79)	0.221		
Gastrointestinal problems	10.93 (9.55)	0.90 (0.44–1.82)	0.769		
Male sexual problems ^2^	18.77 (19.31)	1.60 (1.07–2.40)	0.021		
Defecation problems	16.13 (10.83)	0.85 (0.48–1.51)	0.583		
Weight loss	21.48 (29.85)	1.22 (1.02–1.47)	0.033	1.04 (0.81–1.34)	0.756

^1^ HRs calculated for every 20%, ^2^ Only calculated for male patients, not included in multivariate.

**Table 5:** Overall survival of age-adjusted hazard ratios for change QLQ C30 and QLQ CR38.

		Univariate		Multivariate	
Risk Factor ^1^	Deterioration	HR (95% CI)	*p*-Value	HR (95% CI)	*p*-Value
*QLQ C30*					
Physical function	No Yes	1.09 (0.33–3.64)	0.883		
Role function	No Yes	0.78 (0.23–2.60)	0.682		
Emotional function	No Yes	0.95 (0.40–2.23)	0.902		
Cognitive function	No Yes	0.54 (0.22–1.34)	0.185		
Social function	No Yes	0.61 (0.27–1.40)	0.246		
Global health	No Yes	1.40 (0.66–2.97)	0.376		
Fatigue	No Yes	0.52 (0.24–1.11)	0.09		
Nausea and vomiting	No Yes	0.90 (0.39–2.08)	0.801		
Pain	No Yes	0.82 (0.38–1.73)	0.593		
Dyspnea	No Yes	0.64 (0.25–1.64)	0.35		
Insomnia	No Yes	0.93 (0.43–2.02)	0.857		
Appetite loss	No Yes	0.53 (0.24–1.17)	0.115		
Constipation	No Yes	1.85 (0.81–4.21)	0.144		
Diarrhea	No Yes	0.44 (0.20–0.98)	0.044	0.34 (0.13–0.93)	0.035
Financial difficulties	No Yes	1.50 (0.69–3.29)	0.309		
*QLQ CR38*					
Body Image	No Yes	0.56 (0.26–1.19)	0.13		
Sexual function	No Yes	3.60 (1.36–9.57)	0.01	4.05 (1.47–11.11)	0.007
Future prospects	No Yes	0.60 (0.18–2.02)	0.41		
Micturition problems	No Yes	1.04 (0.48–2.26)	0.923		
Chemo side effects	No Yes	0.83 (0.39–1.80)	0.642		
Gastrointestinal problems	No Yes	0.56 (0.26–1.20)	0.133		
Male sexual problems1	No Yes	0.82 (0.27–2.52)	0.733		
Defecation problems	No Yes	1.84 (0.57–5.88)	0.307		
Weight loss	No Yes	0.33 (0.14–0.77)	0.01	0.32 (0.10–0.97)	0.044

^1^ calculated only for male patients.

## Data Availability

Data are available from the authors upon reasonable request.

## Abbreviations

PROs	patient reported outcomes
HRQoL	health related quality of life
QoL	quality of life
OS	overall survival
EORTC	European organization for research and treatment of cancer
QLQ	quality of life questionnaire
